# A Tuned Microwave Resonant System for Subcutaneous Imaging

**DOI:** 10.3390/s23063090

**Published:** 2023-03-13

**Authors:** Sen Bing, Khengdauliu Chawang, Jung-Chih Chiao

**Affiliations:** Electrical and Computer Engineering, Southern Methodist University, Dallas, TX 75205, USA

**Keywords:** subcutaneous imaging, breast cancer, tumor, nondestructive evaluation (NDE), tuned, phased array antenna

## Abstract

A compact and planar imaging system was developed using a flexible polymer substrate that can distinguish subcutaneous tissue abnormalities, such as breast tumors, based on electromagnetic-wave interactions in materials where permittivity variations affect wave reflection. The sensing element is a tuned loop resonator operating in the industrial, scientific, and medical (ISM) band at 2.423 GHz, providing a localized high-intensity electric field that penetrates into tissues with sufficient spatial and spectral resolutions. The resonant frequency shifts and magnitudes of the reflection coefficients indicate the boundaries of abnormal tissues under the skin due to their high contrasts to normal tissues. The sensor was tuned to the desired resonant frequency with a reflection coefficient of −68.8 dB for a radius of 5.7 mm, with a tuning pad. Quality factors of 173.1 and 34.4 were achieved in simulations and measurements in phantoms. An image-processing method was introduced to fuse raster-scanned 9 × 9 images of resonant frequencies and reflection coefficients for image-contrast enhancement. The results showed a clear indication of the tumor’s location at a depth of 15 mm and the capability to identify two tumors both at the depth of 10 mm. The sensing element can be expanded to a four-element phased array for deeper field penetration. Field analysis showed the depths of −20 dB attenuation were improved from 19 to 42 mm, giving wider coverage in tissues at resonance. Results showed that a quality factor of 152.5 was achieved and a tumor could be identified at a depth of up to 50 mm. In this work, simulations and measurements were conducted to validate the concept, showing great potential for subcutaneous imaging in medical applications in a noninvasive, efficient, and lower-cost way.

## 1. Introduction

Traditional nondestructive evaluation (NDE) methods [1,2,3,4], including computed tomography (CT), X-ray imaging, magnetic resonance imaging (MRI), and ultrasonic measurements, have been widely used for noninvasive cancerous tissue detection, such as breast tumor screening. However, the current instruments are bulky, expensive, and have potential risks. X-ray mammography screening is the mainstay for breast imaging [5,6], but it has been limited by discomfort, the high costs of the instruments, and a shortage of trained radiologists. The high false negative and recall rates require additional imaging and biopsies [7,8,9], further increasing patients’ stress and financial burden. Frequent X-ray exposure may create additional risks. To increase screening accuracy, magnetic resonance imaging (MRI) has been used to provide a second modality of imaging to reduce the rate of false negatives [10], especially for those at high risk. The instrument costs and operation requirements are higher than those of mammography. Due to the limited instruments, potential patients need to travel. They are often unavailable in rural areas where economically disadvantaged groups which are disproportionally high-risk exist. Such inconvenience and complexity increase the possibility of behavioral delay [11] in the potential cancer-patient population for periodic screening, which reduces the chances of identifying pre- or early-stage cancer tissues. Once the tumor is established, the possibility of cancer-cell migration and metastasis increases significantly. Metastasis dramatically reduces the patient survival rate and could be avoided if a timely diagnosis and early treatments are applied [12,13].

Due to the inconsistency in distribution and high contrast in dielectric properties between normal and malignant tissues [14,15,16,17,18,19], electromagnetic waves provide means for noninvasive probing of biochemical properties in tissues. Nonionizing radiation does not have enough energy to damage tissues, unlike X-rays. The microwave electronic components and integrated circuits are readily available and affordable. The dielectric contrasts, electromagnetic propagation, and interference of abnormal tissues can indicate their boundaries. Microwave scattering, including reflection and transmission, measurements have been used to detect breast tumors noninvasively [6,20,21,22]. Such imaging techniques utilizing scattering parameters to reconstruct images include tomography-based passive methods [23,24] and radar-based active methods [25,26,27,28,29]. Recently, microwave-acoustic imaging systems that use microwave pulses to create thermal effects and induce acoustic pressure waves across tissues have been demonstrated [30,31,32,33]. These methods hold great promise for another sensing modality to detect abnormal tissues. However, they still require significant effort in instrumentation because the wave-scattering environments are complex. Methods with various forms of illumination, time-delay estimation of signals, space-time beamforming, adaptive radar synthesis, and short-pulse high-power microwave pulses have been utilized to overcome the challenges of modeling and detecting wave scattering in complicated biological environments. Image reconstruction for each method also requires sophisticated algorithms for beam pattern and multi-path time delay correction and calibration.

Microwave resonators can potentially be used for subcutaneous breast cancer screening. The interaction of electromagnetic fields with tissues provides the potential for noninvasive and high-sensitivity sensing. The resonant frequency and magnitude changes in the reflection coefficients can be evaluated to distinguish tissue types. Resonators typically have smaller footprints compared to bulky instruments, such as X-ray tomography and MRI machines. Resonating cavities or dielectric resonators do not have suitable mechanical configurations to interface with the skin. Planar circuits or antenna resonators are more appropriate, and recent advances in circuit and antenna fabrication on flexible substrates make them able to be worn on the skin. However, the poor resonance at microwave frequencies in most planar resonators that can conform to the skin means they fail to provide reliable and sufficiently deep penetration into tissues. The differences in the tissue permittivities among individuals also make impedance tuning difficult. Thus, the performance suffers from low sensitivity. Using a dynamic matching circuit to achieve a high-quality factor is possible but makes it bulky with design constraints. Impedance tuning for each pixel in an image makes the technique time-consuming and increases insertion losses. The technical challenges in such a conformal microwave resonator are to achieve a high-quality factor without sacrificing the features of being planar and small-form factors.

Our preliminary work [34] involved developing a self-tuned method for impedance-matching in planar-loop resonators by embedding a metal pad. The principle is based on the presence of the center pad to provide distributed capacitances by the gap between the loop and metal pad [35] and the mutual inductance [36] between metal strips across the gap owing to coupled magnetic fields. The distance between the loop and the center pad serves to tune the distributed reactances to match the port impedance in the desired frequency range. The resonance becomes significantly improved without changing the overall size of the loop. The tuning principle is similar to the ones for transmission-line impedance tuning, except no additional transmission line is added outside the loop. With the proposed tuning principle, loop resonators can be made into compact forms with high resonance performance, and they provide great advantages for sensing or near-field signal/power coupling. Applications have been investigated for human hydration monitoring [37,38], subcutaneous implant localization, and wireless power transfer [39,40]. This work aimed to develop a subcutaneous tumor screening system based on our tuned loop resonator. Due to their possession of the most critical screening need, breast cancers are targeted in this paper [41].

Breast tumors typically have higher water content than normal tissues, especially adipose tissues [15]. Adipose tissues in the breast secret biochemical growth factors that have been shown to be related to cancer-cell growth [42]. The water contrast can be up to 10:1 [14], and it consequently determines the distinct dielectric property variations between the malignant and normal tissues in the breast [43,44]. Since the tuned resonance is highly susceptible to dielectric property changes, the tuned sensor can be used to noninvasively locate abnormal subcutaneous tissue boundaries for breast tumor screening. When abnormal tissue is underneath the loop, the resonant frequency or reflection coefficient s_11_ will have a noticeable shift or change. Our previous work [39] developed an implant-locating mechanism based on this principle. However, the implant has a corresponding metal loop, which creates a new resonance with the external sensing resonator to emphasize the contrast for its location. In this work, we investigate if the variations and contrasts in the dielectric properties in biological objects have similar effects, and their uses, to create an image of tissues.

## 2. Sensor Design and Simulations

**Figure 1:**
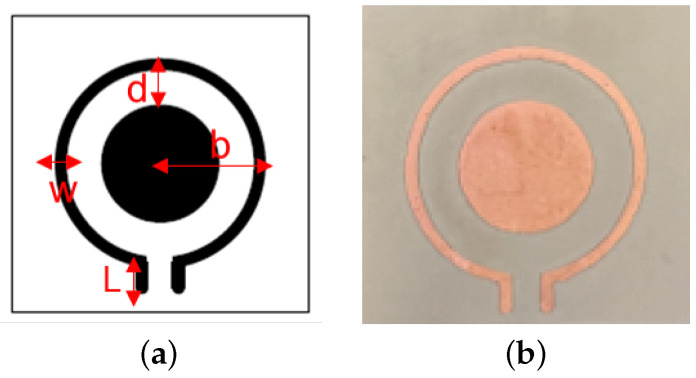
(**a**) The configuration of the tuned sensor: radius *b* = 5.7 mm, loop width *w* = 0.7 mm, stub length *L* = 1.5 mm, tuning gap *d* = 1.95 mm. (**b**) Photograph of the tuned sensor on a flexible polyimide substrate in its flat condition.

**Figure 2:**
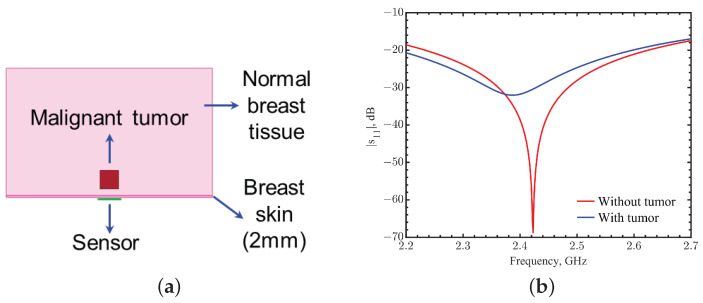
(**a**) Simulation setup. (**b**) Comparison of the magnitudes of reflection coefficients between the cases with and without a tumor inside.

Taking the compact size and sufficient field depth into account, the sensor is designed to function within the ISM (industrial, scientific, and medical) band frequencies at around 2.423 GHz. In the simulations, the model consisted of three parts: skin with a thickness of 2 mm, normal breast tissues, and a malignant tumor in the shape of a cube, 17.1 × 17.1 × 17.1 mm^3^. We assumed first a cubic shape with the same side length as the length of the sensor substrate, so it would be easier to validate with experiments. After validation, tumor and substrate sizes could be varied to investigate details. The sensor was conformed to the breast skin with a distance of 10 mm between the tumor and the sensor. Figure 1 shows the top view of the sensor with its configurations, and Figure 2a shows the simulation setup. Dielectric constants applied in the simulations for breast skin, breast tissues, and tumors were 38, 6, and 59; and the corresponding conductivities were 1.45, 0.13, and 2.8 S/m, respectively, [16,43,44,45,46,47,48,49].

Considering the curvature of the body part, the resonator loop was designed on a flexible polyimide film (DuPont^TM^ Pyralux^®^ FR9220R, Wilmington, Delaware) that could provide firm contact to the skin. The thickness of the film was 76 μm, and it had a dielectric constant of 3.2. The copper’s thickness was 70 μm. The substrate parameters in simulations were the same as the ones in fabrication. The tuned loop configuration is shown in Figure 1a. For an operating frequency of 2.423 GHz for the case without a tumor underneath, the radius of the loop resonator *b* was fixed at 5.7 mm with a connecting stub length *L* = 1.5 mm that was prepared for the measurement connector port. The metal width *w* was 0.7 mm. The spacing *d* between the loop and metal pad was 1.95 mm, tuning the resonance with a reflection-coefficient magnitude of −68.8 dB when no tumor was underneath. A photo of the resonator is shown in Figure 1b. When a tumor was underneath, simulations showed that the resonant frequency shifted to 2.387 GHz with a reflection coefficient of −32 dB, as shown in Figure 2. The distinguishable frequency shift and the change of |s_11_| indicate the possibility of detecting the presence of a tumor.

**Figure 3:**
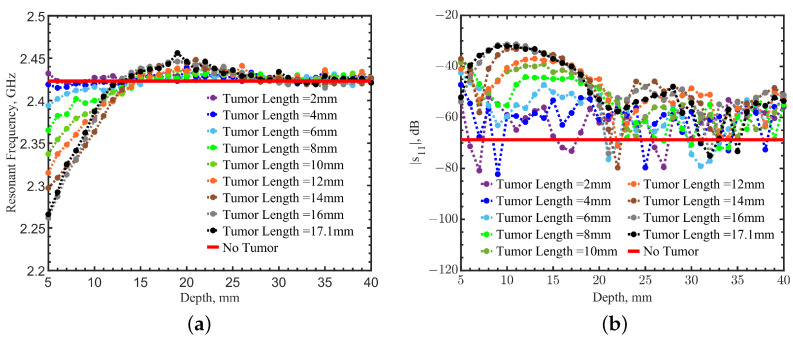
Comparison of simulations with tumor cubic lengths from 2 to 17.1 mm at a depth range from 5 to 40 mm: (**a**) resonant frequencies and (**b**) magnitudes of reflection coefficients.

Based on the same principle, more simulations were conducted with smaller tumors at different depths to further test the sensitivity. The side lengths of simulated tumors varied from 2 to 17.1 mm. The depth was varied from 5 to 40 mm. For each simulated configuration, resonant frequency and its corresponding magnitude of reflection coefficient were collected and plotted in Figure 3a,b, compared to the case without a tumor (solid red line). From the perspective of resonant frequency shift, the tumor became undetectable when the tumor side length was smaller than 4 mm for the depth range of 5–40 mm. The resonant frequency shifted below 2.423 GHz for a tumor depth within 14 mm. The resonant frequencies shifted to higher ones above 14 mm. Resonant frequencies eventually became the same as the ones without a tumor when a tumor was deeper than 25 mm. Similarly, the magnitudes of reflection coefficients did not change monotonically with increasing depth. This phenomenon is likely due to the fact that the electrical fields from the loop were not uniformly distributed or linearly decayed throughout the depth underneath the sensor. Such field distributions change the impact of the tumor permittivity on the effective permittivity experienced by the resonator, inevitably affecting the resonance in the loop. Both information from resonant frequencies and magnitudes of reflection coefficients, however, provide redundancy, so they can be used together to determine the presence of the tumor. Combining both factors may make the detection clearer. A weighted image-fusion method will be introduced later in this work.

## 3. Tumor Localization and Imaging

### 3.1. Raster Scan

**Figure 4:**
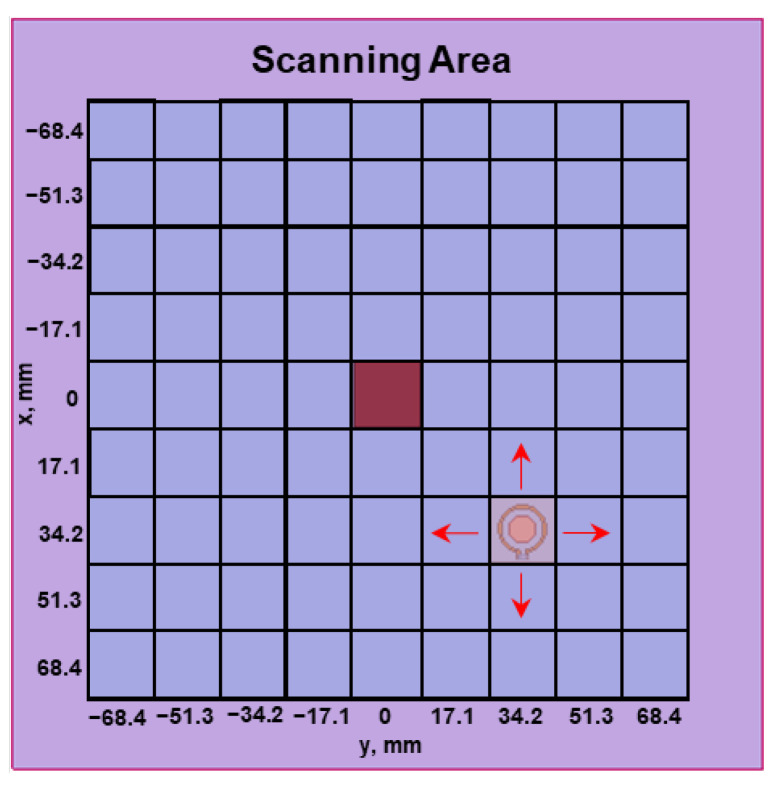
Schematic for the raster scan. A tumor with a volume of 17.1^3^ mm^3^ was positioned at a depth of 10 mm and (x = 0, y = 0). In the illustration, the sensor is (34.2, 34.2) to obtain the reflection coefficient of the pixel (34.2, 34.2).

In applications, the tumor’s location requires a "heatmap" with a 2-dimensional raster scan of the targeted tissue area by moving one sensor. The image can also be obtained with an array of loop resonators switched temporally to acquire individual pixel data. Based on the reflection coefficients, heatmaps can be generated to illustrate the location of the abnormal tissue. A discrete raster scan was conducted in simulations with the tuned loop to validate this concept. Figure 4 shows the schematic of the simulation setup for the raster scan. A tumor size of 17.1^3^ mm^3^ was placed at (x, y) = (0, 0) at a depth of 10 mm, where the distance from top surface of the tumor to the skin surface was 10 mm. The resonator loop diameter was 11.4 mm. Each pixel was 17.1 × 17.1 mm^2^. The total scanning area was 13.68 × 13.68 cm^2^ with 9 × 9 pixels. The sensor in Figure 4 was at (34.2, 34.2) and would complete the raster scan.

**Figure 5:**
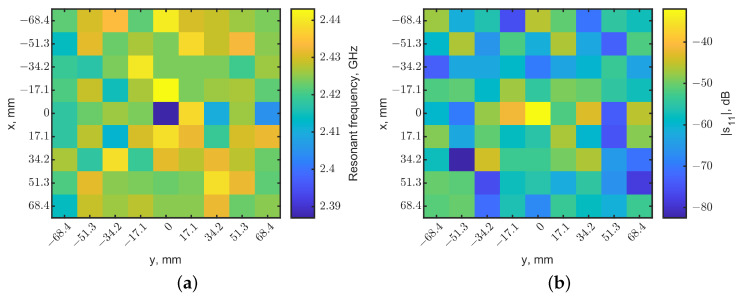
Localization maps generated from the reflection coefficients for the tumor at a depth of 10 mm. The tumor is located at (x = 0, y = 0). (**a**) Map of resonant frequencies, the dark pixel indicates the tumor’s location. (**b**) Map of |s_11_|; the light-yellow pixel indicates the tumor’s location.

**Figure 6:**
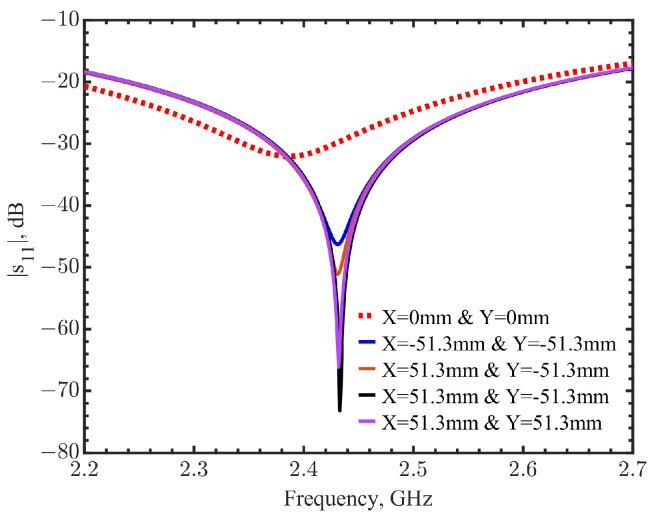
Comparison of reflection coefficients at the center (dashed line) and four near corners (solid line). The tumor is at the center as (x = 0, y = 0).

Figure 5a shows the heatmap of the resonant frequencies, which is represented in pixels with different color scales normalized from 2.387 to 2.443 GHz. It clearly shows that the location of the tumor is at (0, 0), where the resonant frequency is the minimum. The reflection coefficients in the pixels at the four corners of this scanned map are compared with the one in the center, as shown in Figure 6. The spectral curves clearly indicate that the distinguish frequency shifts with or without a tumor underneath can be distinguished. Moreover, the magnitudes of reflection coefficients can further clarify the tumor’s location. When a tumor is directly underneath, the effective permittivity variation makes the tuned loop become untuned, significantly changing |s_11_|. Figure 5b shows the heatmap generated from the magnitudes of reflection coefficients. The light-yellow pixel in the center with a reflection coefficient of −32 dB indicates the tumor’s location, compared to the neighbor pixels with reflection coefficients of around −60 dB. The adjacent pixel and other pixels away from the center show magnitudes in the range of −40 to −50 dB. The increases in |s_11_| were likely due to the resonator field distributions making the heatmap look noisy.

**Figure 7:**
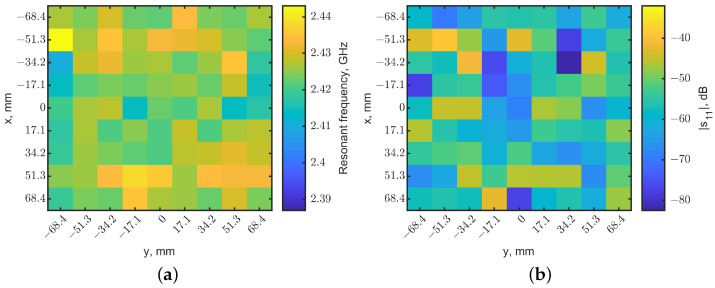
Localization maps generated from (**a**) resonant frequencies and (**b**) reflection-coefficient magnitudes for the case without a tumor. The color scales are the same as the ones in Figure 5.

A raster scan was performed with the same setup without a tumor. The heatmaps of resonant frequencies and reflection-coefficient magnitudes are shown in Figure 7, using the same color scales as Figure 5. Compare Figure 5a and Figure 7a: no dark pixel was in Figure 7a. Similarly, Figure 5b shows clearly the center pixel with a higher reflection coefficient compared to other pixels in Figure 5b and all pixels in Figure 7b.

### 3.2. Weighted Image Synthesis

**Figure 8:**
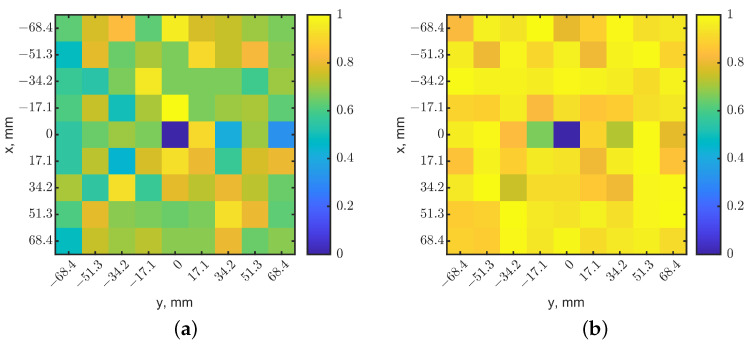
Normalized localization maps from (**a**) resonant frequencies and (**b**) magnitudes of reflection coefficients. The tumor is located in (x = 0, y = 0) at a depth of 10 mm. The dark-blue pixel indicates the tumor’s location.

Unlike using only the frequency shift information in [39], the reflection-coefficient magnitudes may provide a second set of data to reconstruct the image. To combine these two sets of data, resonant frequencies and |s_11_| magnitudes (in ratio instead of dB) of each pixel were normalized to a range from zero to one by their minimal and maximal values in the map. In Figure 5a, the resonant frequency decreased with the presence of a tumor at 10 mm depth, compared to other pixels with no tumor. Therefore, the center pixel became zero after normalization, as its resonant frequency was the lowest on the map. On the contrary, due to the detuned resonance, |s_11_| increased, as shown in Figure 5b. The center pixel became closer to one after normalization because it has the largest value. A linear transformation converted the normalized |s_11_| map to match the indication in the resonant frequency map. Figure 8a,b show the normalized heatmaps of resonant frequency and |s_11_|, respectively. The colors clearly indicate the tumor’s location. However, Figure 8 shows a special case with specific tumor size and depth. According to Figure 3a,b, resonant frequencies could increase when tumor sizes vary or at other depths, and reflection-coefficient magnitudes decrease compared to those without tumors. The same linear transformation is applied to the normalized resonant frequency map or |s_11_| map as one sees fit to have consistent scale maps.

Although to human eyes, the heatmaps in Figure 8 seem obvious and easily identify the tumor’s location, comparing the heatmaps may be tedious and subjective if the images are noisy. We further fused the heatmaps together with a weighting factor. Figure 3a,b show that changes of |s_11_| are more sensitive than the resonant frequency shifts when a tumor is in a shallow depth above 14 mm. Additionally, as expected, |s_11_| images were found to become noisy, even when the deep tumor was directly underneath the sensor. The normalization further magnified the noises in |s_11_|, making it difficult to recognize the tumor’s location. A weighting factor *W* was applied to the normalized resonant frequency matrix $M_{f}$ and the |s_11_| matrix $M_{r}$ to construct a heatmap $M_{s}$ as:

(1) $$M_{s}=M_{f}\times W+M_{r}\times \left(1-W\right)$$

**Figure 9:**
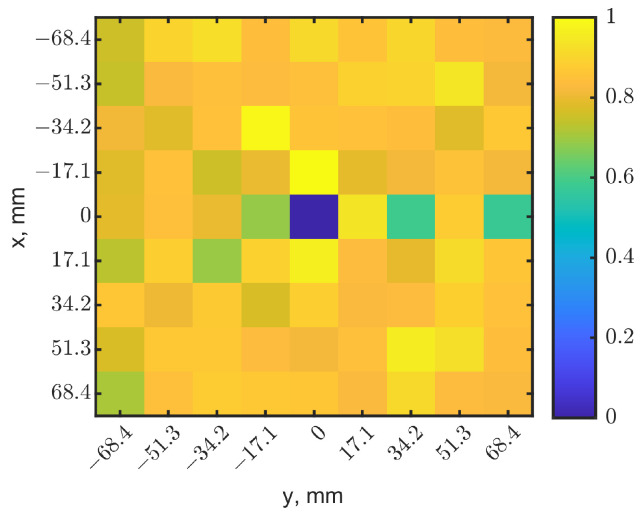
Synthesized heatmap with a weight *W* = 0.5 in the normalized resonant frequencies and |s_11_|. The dark-blue pixel indicates the tumor’s location.

The weighted images provide an option for evaluating the significance between resonant frequencies and the magnitudes of reflection coefficients at specific depth ranges. Due to the unknown and unpredictable electromagnetic field distributions inside the tissues, which are person-dependent and physiologically variable, the weighted images can help to highlight the contrasts. A proper *W* can enhance the image quality, and in return, increase the detection depth. *W* was set as 0.5 as the default and could be changed according to the image quality. Figure 9 shows the constructed image with *W* = 0.5. The dark pixel in the middle clearly shows the tumor’s location. The pixel magnitude in the center had a scale of 0, and the eight neighbors had scales of 0.69–1. Compared to Figure 5a,b, the synthesized image has a better contrast for the pixel with a tumor underneath, and the single fused image provides a more convenient means for image recognition.

**Figure 10:**
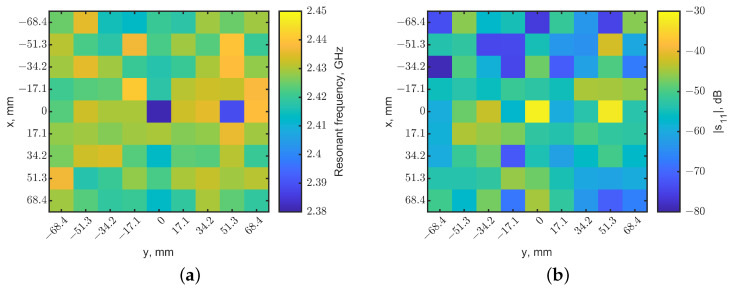
Heatmaps generated from reflection coefficients for two tumors at a depth = 10 mm. The tumors were located at (x, y) = (0, 0) and (0, 51.3). (**a**) Map of resonant frequencies; the dark-blue pixel indicates the tumor’s location. (**b**) Map of |s_11_|, the light-yellow pixel indicates the tumor’s location.

**Figure 11:**
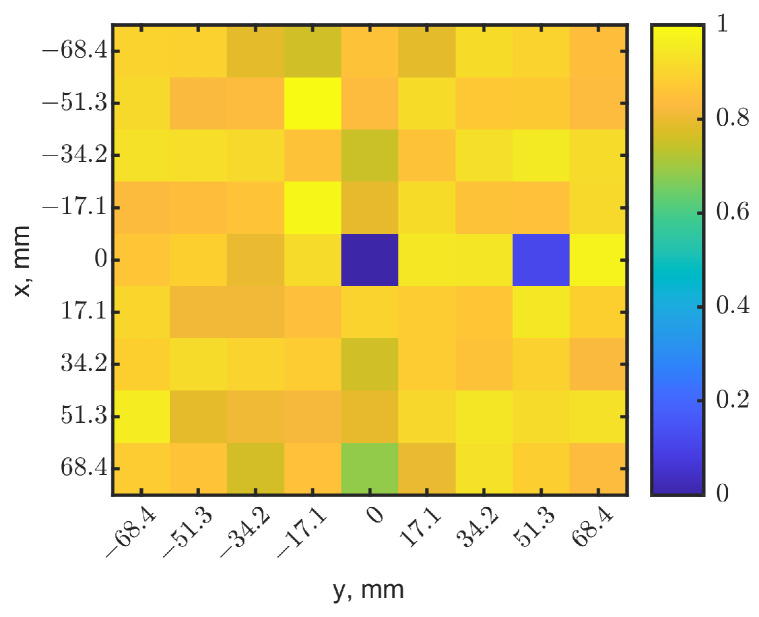
Synthesized heatmap for two tumors at (x = 0, y = 0) and (x = 0, y = 51.3). The dark-blue pixel indicates the tumor’s locations.

To test the method, two identical tumors with the same cubic dimensions were placed at (x, y) = (0, 0) and (0, 51.3), both at a depth of 10 mm. The same 2-dimensional raster was conducted with a 9 × 9-pixel resolution. Figure 10a,b show the heatmaps generated from the resonant frequencies and reflection-coefficient magnitudes, respectively. With the same approach implemented for Figure 9, the resonant frequencies and reflection-coefficient magnitudes were normalized and then fused to create a 9 × 9 image. Figure 11 shows the synthesized image with *W* = 0.5. It clearly identifies the locations of two tumors in the dark pixels. Compared to the raw images in Figure 10, the synthesized image has better contrasts, as expected, further validating the method. 

## 4. Measurements

The tuned loop was fabricated on the flexible polyimide film (DuPont^TM^ Pyralux^®^ FR9220R). The metal pattern was etched after photolithography was applied with a photomask on the photoresist-covered film. Figure 1b shows a photo of the fabricated resonator. The parameters and dimensions in the fabricated devices are identical to those in simulations. The resonator was connected with a 50-Ω adaptor to a vector network analyzer (Keysight PNA N5227B).

**Figure 12:**
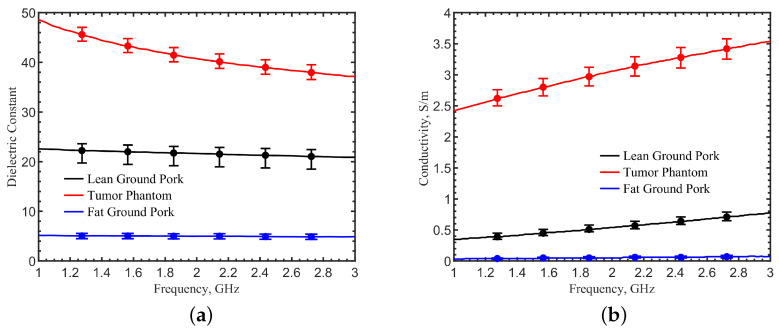
Measurements of dielectric properties: (**a**) dielectric constant and (**b**) conductivity for lean ground pork, tumor phantom, and fat ground pork, which mimic the breast skin, normal breast tissue, and breast tumor in experiments. The error bars were obtained from 5 measurements with respect to averaged values.

**Figure 13:**
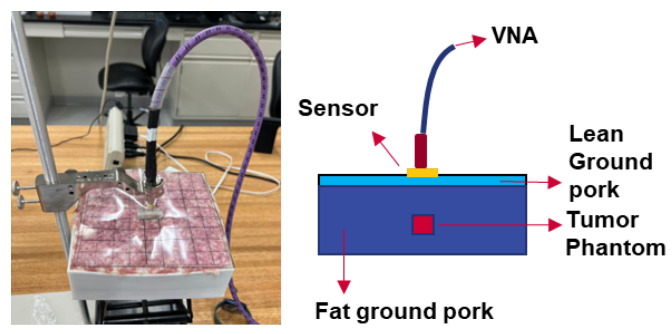
Setup of the subcutaneous imaging measurements.

**Figure 14:**
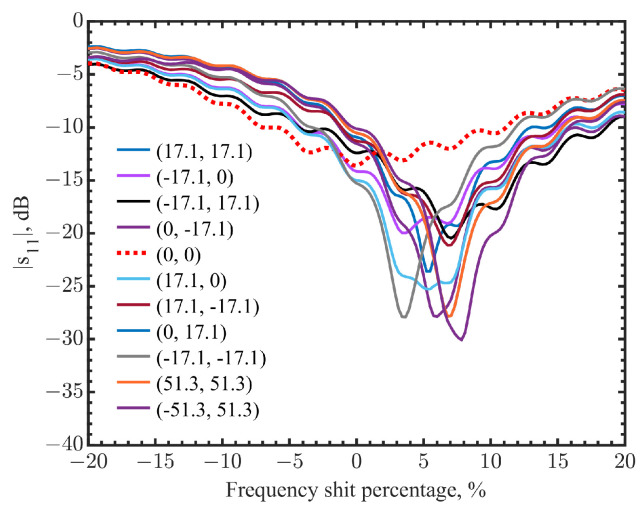
Comparison of normalized frequency shifts in measurements at the center (dashed line), the neighbor pixels, and two pixels at the corners (solid lines). The pixel location is indicated as (x, y) on the map, and the tumor is at the center as (0, 0).

In the literature, there are some discrepancies in dielectric properties. Generally speaking, high dielectric contrasts were found between malignant tissues and normal tissues [16,43,44,45,46,47,48,49]. However, a high contrast was observed between malignant tissues and adipose-dominated tissues, up to a 10:1 ratio, but it became much lower between malignant tissues and fibroconective/glandular tissues, which have high water content [44]. Therefore, it should be noted that a high-contrast tumor phantom model was chosen, mimicking the specific adipose-dominated cases mentioned in the literature, to validate the concept and detection feasibility. Ground pork mixed with fat and lean ground pork was used to mimic breast tissues and skin. Artificial silicone- and carbon-based materials [14], mixed to reach targeted permittivities, were selected to mimic the documented tumor dielectric properties in simulation. Dielectric properties measurements for these phantom tissues were conducted using a coaxial probe kit (Keysight N1501A). The measured dielectric constants and conductivities are shown in Figure 12a,b. Each dataset with the error bars was obtained from five measurements at five locations, with spacing at least 5 cm apart to avoid sensing overlapped volumes [50]. Average values were then obtained. Discrepancies were observed between the measurements in phantoms and the simulations that utilized the documented dielectric parameters [16,43,44,45,46]. This was expected because the documented dielectric parameters in the literature were obtained from tissues removed from the human body, in small volumes and with loss of water. The contrasts in dielectric properties between normal and malignant tissues, however, were kept consistent with our phantoms. The phantom materials were packed into a cubic box of 19 × 19 × 12 cm^3^ to mimic the body part. The measurement configuration was the same as the one in the simulations. Figure 13 shows the measurement setup. The tumor cube was inserted at the center point (0, 0) at a depth of 10 mm. The phantom sides were protected from water evaporation. The sensor scanned the phantom surface with a step spacing of 17.1 mm. The results of the 3 × 3 pixels in the center were compared with the two pixels at the corners (51.3, 51.3) and (−51.3, 51.3) of the phantom in Figure 14. The frequencies were normalized by the resonant frequency at the center point (0, 0), showing shifts of about 3.66–6.79% for the neighbor pixels and 7.05–7.83% for the two corner pixels that did not have a tumor underneath. The ripples in the frequency spectrum were likely due to the standing waves between the sensor adaptor and the skin, as applied pressures on the cable changed the ripple magnitudes.

**Figure 15:**
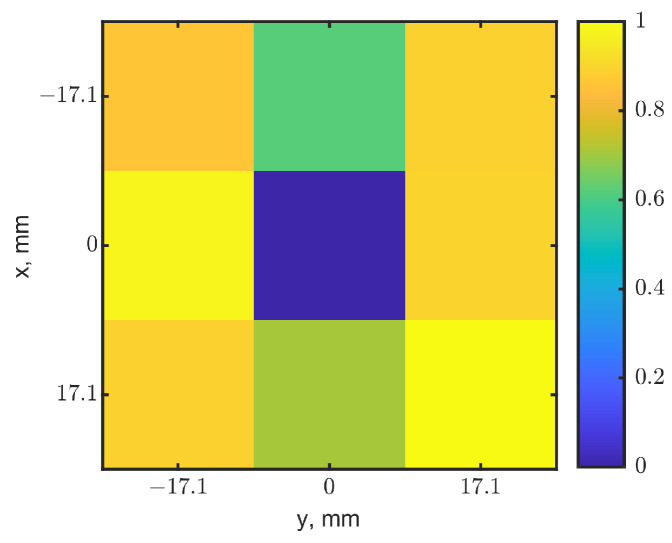
Heatmap from measurements with a tumor of 17.1^3^ mm^3^ located at (0, 0) with a depth of 10 mm.

Frequency shifts and reflection-coefficient magnitudes were normalized, and their maps were fused with the aforementioned method. Figure 15 shows the synthesized heatmap with a weighting factor of 0.5. The tumor’s location was clearly identified. Other pixels outside the 3 × 3 pixels are similar to the surrounding pixels, so they are not shown. The eight neighbor pixels had both resonant frequencies and reflection-coefficient magnitudes different from the center pixel where the rumor located, shown with the spectral curves in Figure 14. The normalization and fusion of both maps further enhanced the image, as the center close to the scale zero and other pixels above 0.6. Simulation and measurement results match well, and they validate the imaging method of a raster scan using the tuned loop resonator.

## 5. Phased Array Loops

### 5.1. Design and Validation

**Figure 16:**
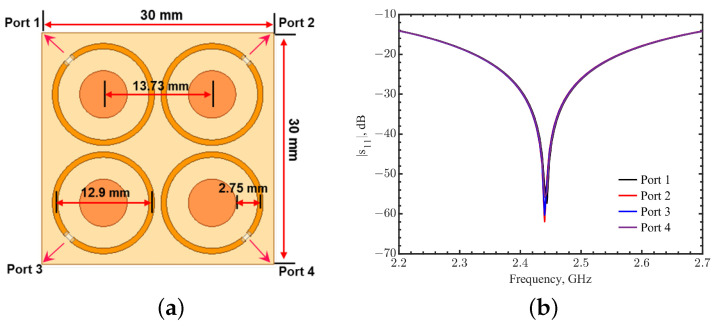
(**a**) The phased array loop structure consisting of four tuned loops in a mirrored configuration. The phase difference was 180${}^{\circ }$ between the neighbor elements. (**b**) Simulations of reflection coefficients for ports 1–4 in the tuned phased loop array.

Typical normal and nonlactating female human breast tissue thickness is around 50 mm [51,52,53]. Nearly 50% of breast tumors occur in the quadrant close to the armpit [54], where the thickness of the breast is less than 25 mm. Considering a mildly compressed breast spanning between the skin surface and rib cage, the sensor needs to be able to detect a depth of at least 30 mm. To increase the field depth, four tuned loops were placed in a 2 × 2 array with a mirrored configuration to form better field coverage in the tissues. The spacing between the loop centers was 13.73 mm, and the ports faced outward, as shown in Figure 16a. The radius *b* for each loop was 6.1 mm with a tuning gap spacing *d* = 2.75 mm. Four 50-Ω lumped ports had a phase difference of 180${}^{\circ }$ between the neighbor elements. Figure 16b shows the resonance in these four ports at 2.44 GHz. The quality factor, defined as the center frequency divided by the 3-dB bandwidth, was 152.5.

**Figure 17:**
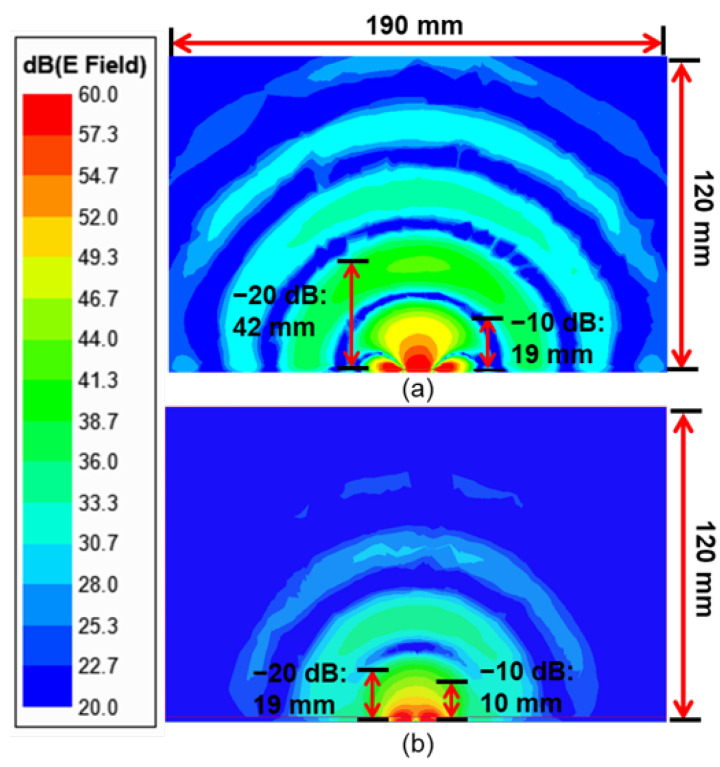
Electric fields in the vertical cross-section plane into the tissue for (**a**) the tuned phased loop array and (**b**) the single tuned loop resonator.

In the tuned-loop-array configuration, the combined electrical fields penetrate deeper into the tissues. The field distribution in a 2D cross-section inside the tissue is shown in Figure 17a, compared to the fields in the same plane for a single-tuned-loop resonator in Figure 17b. With higher intensities of electric fields, the effective permittivity experienced by the resonator has a more pronounced effect on the resonance. This effect makes the object at the same depth contribute more to the effective permittivity, so it can help sense a smaller size of the tumor. The dielectric property at a deeper depth also has more impact on the effective permittivity because the fields reach deeper. Comparing the field distributions, the −10-dB and −20-dB attenuation depths increased from 10 and 19 mm to 19 and 42 mm for the arrayed configuration, respectively. A raster scan was conducted for a tumor of 17.1^3^ mm^3^ at a depth of 50 mm. The tumor dimensions were the same as the ones in previous simulations with a single loop for comparison. The pixel size was set as 30 × 30 mm^2^, the same as the dimensions of the array substrate. In each scan, the resonant frequencies and reflection-coefficient magnitudes at ports 1–4 were averaged first before normalization. Then, the fused image matrix was obtained with Equation (2), where *i* = 1–4 for the specific port:

(2) $$M_{s}=\frac{1}{4}\sum M_{fi}\times W+\frac{1}{4}\sum M_{ri}\times \left(1-W\right)$$

**Figure 18:**
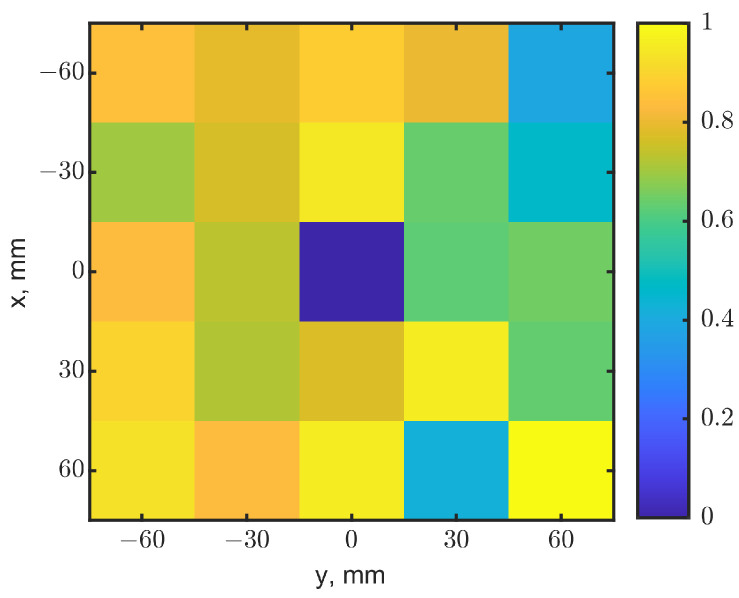
Heatmap by the tuned phased loop array. The tumor was placed at (0, 0) with a depth of 50 mm and had a size of 17.1^3^ mm^3^. The dark-blue pixel indicates the tumor’s location.

Figure 18 shows the fused heatmap with 5 × 5 pixels from the normalized resonant frequencies and reflection-coefficient magnitudes with a weighting factor of 0.5. The dark pixel clearly identifies the tumor’s location in Figure 18. With the same tumor and weighting factor, the array can detect the tumor at a depth of 50 mm, compared to the 25 mm depth by a single loop.

**Figure 19:**
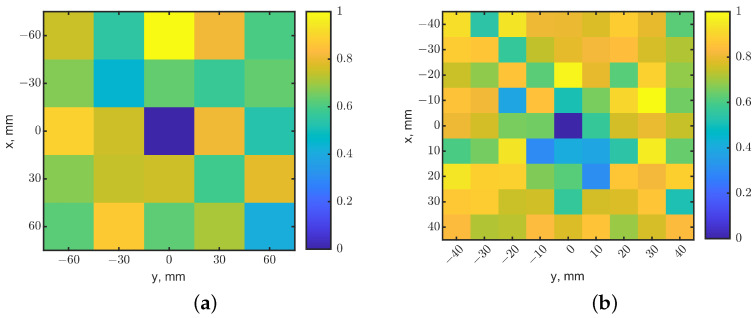
Comparison of heatmaps by the tuned phased loop array with different raster step sizes of (**a**) 30 and (**b**) 10 mm. The tumor was placed in (0, 0) at a depth of 25 mm and had a smaller size of 10^3^ mm^3^. The dark-blue pixels indicate the tumor’s locations.

To test the ability to sense a smaller tumor, another raster scan was conducted with a tumor of 10 × 10 × 10 mm^3^ at a depth of 25 mm. The raster scan had a step of 30 mm. Figure 19a shows the fused heatmap and correctly indicates the tumor’s location. To identify the tumor size, an oversampling scan was repeated with a scan step of 10 mm. The dark pixel clearly indicates the tumor’s location with a better resolution of the tumor size, as shown in the 9 × 9-pixel fused heatmap of Figure 19b. This shows that the array can be used, similarly to the principles of synthetic aperture radar [55], with oversampling techniques and related image processing algorithms to increase spatial resolutions at a deeper depth in tissues.

### 5.2. Radiation Direction Steering

**Figure 20:**
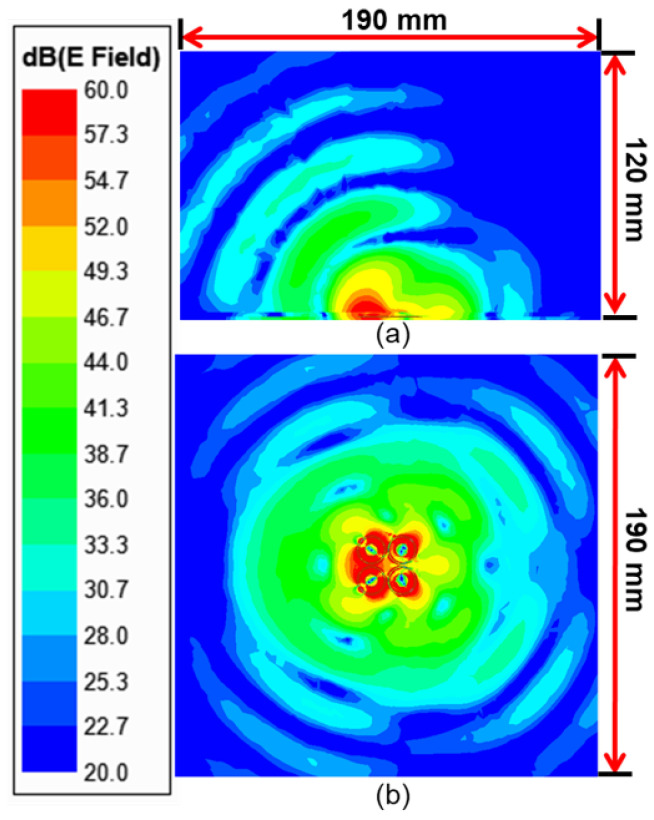
Cross-sections of electric field distributions of the tuned phased array loop resonator with a phase difference of 90${}^{\circ }$. (**a**) Side view and (**b**) top view.

In the loop array structure, the phase difference was fixed at 180${}^{\circ }$, which concentrated the electric fields and penetrated deeper into tissues underneath the center of the array. In the finite-element simulations, we found that the radiation direction of the electrical field could be steered with different phase shifts. For instance, the electric fields radiated in a certain direction when the phase difference of 90${}^{\circ }$ between adjacent loops was set, shown in the cross-section and top views of Figure 20. This feature suggests that the phased loop array can change the sensing area without physically moving its location. The change in field distributions in a certain direction highlights the contribution of tissue permittivities in the effective permittivity experienced by the resonator.

**Figure 21:**
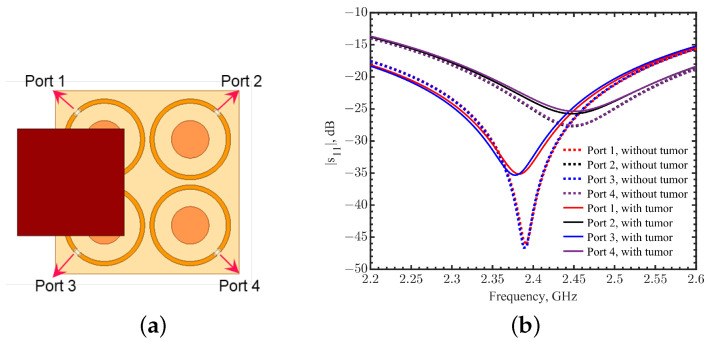
(**a**) Setup to investigate the impact of steered fields by changing the phase difference in the array. A tumor was placed off-center, symmetrically but partly underneath loops 1 and 3 at a depth of 10 mm. (**b**) Comparison of reflection coefficients at ports with and without a tumor underneath the array.

For verification, simulations with and without a tumor underneath the skin were conducted using the phased array with a phase shift of 90${}^{\circ }$. Dashed and solid lines indicate two sets of resonance in four ports, shown in Figure 21b. Without a tumor, a frequency shift of 56 MHz between ports 1 and 3 and ports 2 and 4 was observed. The same phenomenon can be found in [24] due to the field coupling between adjacent metal patterns. When the field distributions are changed, steered in this case underneath ports 1 and 3, the distributed capacitance and mutual inductance inevitably affect the resonances in the loops. From Figure 20, the field distribution changes occur similarly to ports 1 and 3. Ports 2 and 4 also experience the opposite changes. Ports 1 and 3 have stronger field coverages compared to ports 2 or 4, presenting more effects on the effective permittivities of ports 1 and 3. As a result, the resonance frequency shifts by 56 MHz in these two ports. A tumor of 17.1^3^ mm^3^ was placed off-center, symmetrically but partly covering loops 1 and 3, at a depth of 10 mm, as shown in Figure 21a. The solid curves in Figure 21b show the frequency shifts in ports 1 and 3 from 2.392 to 2.378 GHz, with |s_11_| changed from −46.5 to −35.3 dB, while the reflection coefficients for ports 2 and 4 nearly stay the same. The noticeable frequency shifts and |s_11_| magnitude changes can be used to indicate the presence of the tumor in a specified direction under the skin. Without moving the arrayed sensors, nearby areas can be scanned with phase changes in ports to highlight the direction or area of interest before a finer-resolution scan to find the tumor size and depth.

## 6. Discussion

**Figure 22:**
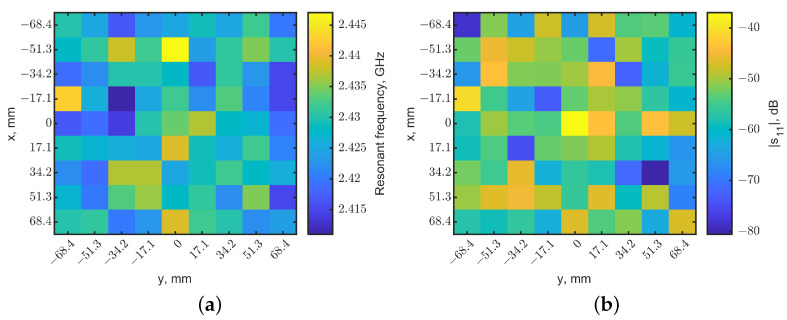
Heatmaps generated from reflection coefficients for a tumor at a depth = 15 mm. The tumor was located at (x = 0, y = 0). (**a**) Map of resonant frequencies and (**b**) map of the magnitudes of reflection coefficients.

**Figure 23:**
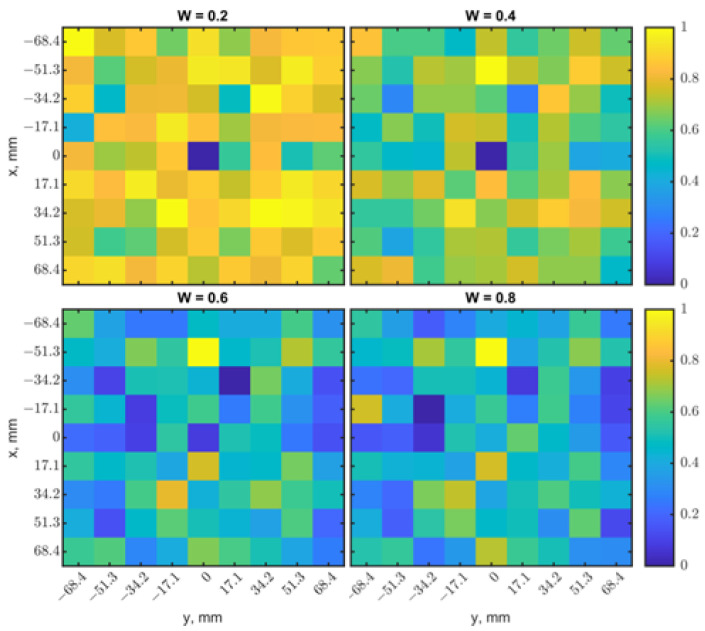
Comparisons of heatmaps fused with normalized results in Figure 22 and with different weighting factors *W* of 0.2, 0.4, 0.6, and 0.8.

**Figure 24:**
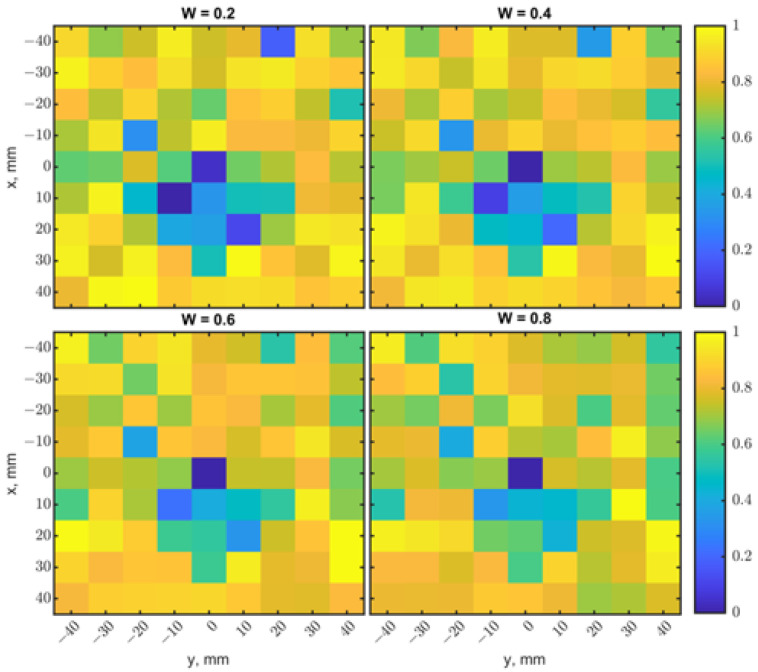
Comparisons of heatmaps fused from the results in Figure 19 with different weighting factors *W* of 0.2, 0.4, 0.6, and 0.8. The case of *W* = 0.8 produces a better contrast with which to locate the tumor.

The weight factor *W* was fixed at 0.5 as a default for both the single loop and loop array, providing performance comparison with different variables. However, a proper *W* can increase the image quality and detection depth by enhancing contrasts. To demonstrate this idea, raster-scan simulations were conducted with a tumor of 17.1^3^ mm^3^ placed at (0, 0) at a depth of 15 mm. Figure 22a shows the heatmap generated from the resonant frequencies. The scanning result was blurry with noises. The heatmap of |s_11_| shows a better ability to identify the tumor’s location in Figure 22b, but the image still looks fuzzy. Similarly, Figure 3 shows that |s_11_| changes are distinguishable at a depth range of 13–15 mm, and it is not easy to identify the locations with their resonant frequencies. In such a case, normalized a |s_11_| matrix $M_{r}$ should be emphasized to make the fused image $M_{s}$ have better contrast for location detection. Figure 23 shows fused images with weight factors *W* of 0.2, 0.4, 0.6, and 0.8. The tumor’s location becomes more evident with *W* = 0.2. On the contrary, based on the results in Figure 19b, when different weight factors *W* from 0.2 to 0.8 were applied, as shown in Figure 24, the tumor’s location became more apparent with a weighting factor *W* of 0.8. In practical scenarios, tumor size and depth are unknown, so multiple heatmaps with a range of weighting factors should be generated to find the image with the best contrast. An algorithm for the automation of image generation and classification will be investigated as the next step.

The purpose of this work was to demonstrate the principle and feasibility of utilizing a flexible radio-frequency resonator that provides convenience and comfort as a first-step, quick screening tool to detect abnormal tissues within the skin, noninvasively. The method is different from microwave radar-based and microwave-acoustic imagers that are under research and serves a different purpose. Some assumptions were made in this stage of concept validation. The simulation and measurement models were simplified in order to exclude interference elements in the studies and compare scenarios directly, including pixel sizes and tumor sizes/shapes. Specific fibroglandular tissues were excluded. Fibrogladular tissues consist of fibrous connective tissues; functional glandular tissues, such as milk glands and ducts; and fatty tissues. The compositions of fibroglandular tissues and their densities are related to the risk of breast cancer occurrences [56,57] and should be considered in clinical imaging. In this study, we chose the high-dielectric-contrast cases between malignant and normal adipose-dominated tissues [44] with the assumption of dielectric constants of 6 and 59 (from [16,43,44,45,46,47,48,49], close to the 1:10 contrast observed in [14,44,50]) for normal and tumor tissues, along with their corresponding conductivities of 0.13 and 2.8 S/m to illustrate the principle of noninvasive detection and imaging. According to [50], the contrast ratios of dielectric constants and conductivities between normal and cancer tissues were 63:8 and 4:0.5 at 2 GHz, respectively. The results agreed with those in [44], as the biopsy samples of normal tissues were predominantly adipose tissues with low water content. However, the dielectric constants and conductivities were found to be only 17.5% and 16.2% higher than those in mammary gland tissues at 1.6 GHz [51]. Discrepancies in dielectric properties in biopsy samples were likely due to the volume fractions of cancer cells in the excised tumors, according to [43,51]. When comparing malignant tissues to fibroconnective and glandular tissues that have high water content, their contrasts may dramatically decrease. Therefore, in the next steps, incorporating different compositions of fibroglandular tissues within the models will be investigated by varying the contrast ratios.

## 7. Conclusions

In this work, we demonstrated a new nondestructive evaluation (NDE) method using a tuned radio-frequency resonant loop that can conform to the skin to detect tissue effective permittivity variations under the skin. The detection of breast tumors was selected to demonstrate the concept of subcutaneous sensing. Tissue-mimicking materials and ground pork, with similar dielectric properties to normal and tumor tissues, were used in phantoms for simulations and measurements. Simulations and corresponding measurements verified the feasibility of noninvasive sensing. It was shown that electromagnetic fields were altered in the presence of a tumor phantom due to the high contrast between the tumor and normal breast tissues. The high-quality-factor resonance was able to detect the abnormity. A construction method of fused images from the heatmaps of normalized resonant frequencies and magnitudes of reflection coefficients was introduced. The fused heatmaps highlight the boundaries of the tumor images with a weighting factor. A tuned array of four loops was developed to provide better spatial resolutions to recognize deeper or smaller tumors. Preliminary studies showed its feasibility.

Additionally, by varying phase shifts among resonators, distributions of electromagnetic fields can be modified without moving the sensor and provide a means to scan electrically to identify the area of interest. The tumor’s location and size in the area of interest then can be better identified with oversampling scans. The synthesized images further emphasize the contrast of the image. This work mainly focused on validating the feasibility of subcutaneous tumor imaging. The tumor’s sizes were set up to be close to the sensor’s dimensions for the purposes of illustration and demonstration. The tumor’s sizes may have been relatively larger than the tumors of interest in the realistic cases. Therefore, future works will focus on exploring the sensing capability for smaller tumors. Moreover, potential methods to improve the spatial resolution, such as reducing loop dimensions at a higher operating frequency, scanning with oversampling, optimizing the weighting factor by utilizing machine learning, and increasing the element number in the array, will be investigated.

## Data Availability

The data presented in this study are available on request from the corresponding author.
